# Platelet indices and blood pressure: a multivariable mendelian randomization study

**DOI:** 10.1186/s12959-023-00475-6

**Published:** 2023-03-20

**Authors:** Yuhan Xu, Yijing Guo

**Affiliations:** 1 School of Medicine, Southeast University, Nanjing, Jiangsu Province 210009 China; 2 Department of Neurology, Affiliated ZhongDa Hospital of Southeast University, Nanjing, China

**Keywords:** Mendelian randomization, Blood pressure, Platelet indices

## Abstract

**Background:**

Platelet indices are blood-based parameters reflecting the activation of platelets. Previous studies have identified an association between platelet indices and blood pressure (BP). However, causal inferences are prone to bias by confounding effects and reverse causation. We performed a Mendelian randomization (MR) study to compare the causal roles between genetically determined platelet indices and BP levels.

**Methods:**

Single-nucleotide polymorphisms (SNPs) associated with platelet count (PLT), plateletcrit (PCT), mean platelet volume (MPV), platelet distribution width (PDW), and BP at the level of genome-wide significance (p < 5 × 10^− 8^) in the UK Biobank were used as instrumental variables. In bidirectional univariable MR analyses, inverse variance-weighted (IVW), MR‒Egger, and weighted median methods were used to obtain estimates for individual causal power. In addition, heterogeneity and sensitivity analyses were performed to examine the pleiotropy of effect estimates. Finally, multivariable MR analyses were undertaken to disentangle the comparative effects of four platelet indices on BP.

**Results:**

In the univariable MR analyses, increased levels of PLT and PCT were associated with higher BP, and PDW was associated with higher DBP alone. In the reverse direction, SBP had a minor influence on PLT and PCT. In multivariable MR analysis, PDW and PLT revealed an independent effect, whereas the association for PCT and MPV was insignificant after colinear correction.

**Conclusion:**

These findings suggest that platelets and BP may affect each other. PDW and PLT are independent platelet indices influencing BP. Increased platelet activation and aggregation may be involved in the pathogenesis of hypertension, which may provide insights into evaluating thromboembolic events in people with high BP. The necessity of initiating antiplatelet therapy among hypertension groups needs further investigation.

**Supplementary Information:**

The online version contains supplementary material available at 10.1186/s12959-023-00475-6.

## Introduction

High blood pressure (BP) is a significant risk factor for cardiovascular diseases (CVDs) [1–4]. It has been widely acknowledged that the main complication of elevated BP is related to thrombosis rather than haemorrhage [5, 6]. Therefore, the role of platelet activation in CVD has led us to speculate that it may be associated with BP [7, 8]. Previous studies confirmed that increased platelet activation and aggregation are involved in hypertension, and platelets seem to be more hyperactivated in those with elevated BP [9–11]. Thus, recommendations are reasonable to combine acetylsalicylic acid with antihypertensive therapy in individuals at high risk of CVD under well-controlled BP and careful assessment of bleeding events [12]. However, whether and when to initiate antiplatelet therapy is under debate because it has not been shown to modify mortality for primary prevention, despite the use of antiplatelet therapy in secondary CVD prevention being incontrovertible [13]. Notably, an increased bleeding risk may counterbalance the potential benefits of CVD prevention, but most importantly, the causal link between them is unclear. If exposure has a noncausal association with an outcome, then exposure-targeted therapies are unlikely to have any real benefit.

In clinical practice, platelet count (PLT), plateletcrit (PCT), mean platelet volume (MPV), and width of distribution (PDW) are leading platelet indices, which can be used to reveal the biochemical and functional changes of the platelet [24]. Therefore, evaluations of platelet indices that indicate platelet bioactivity could be vitally crucial for monitoring hypertension occurrence and progression. Prior observational studies have documented that high platelet indices, such as PLT, predispose people to hypertension [14–16]. However, constrained by potential methodological limitations, such as residual confounding and reverse causation, traditional observational study designs cannot infer causality concerning the role of platelets in the development of hypertension. An alternative approach is the Mendelian randomization (MR) design, which utilizes genetic variants as instrumental variables (IVs) for an exposure to determine the causality of an exposure-outcome association [17, 18]. Previous MR research has elucidated the causal relationship between haematological traits and BP as well as other diseases [19–22]. However, there is only one MR study assessing the relationship between PLT and BP, and PLT on its own was deemed insufficient because the platelet indices were correlated [23, 24]. Therefore, it is plausible to assume that each trait played an individual causal role or that one or more predominated and accounted for the associations of related characteristics. Given correlations across platelet traits, the multivariable MR (MVMR) framework, as an extension to the traditional MR method, should be recommended to simultaneously appraise the association of correlated multiple risk factors with the outcome of interest [25, 26]. With the inclusion of genetic associations for various exposures in the same model, MVMR can evaluate which traits retain causal associations with the outcome through genetic protection from conventional bias, including unobserved confounding, reverse causation, and collider bias [27]. In the present study, we employed bidirectional MR analysis to determine associations of individual platelet indices with BP and then MVMR analysis to disentangle the comparative effects of four platelet indices.

**Fig. 1:**
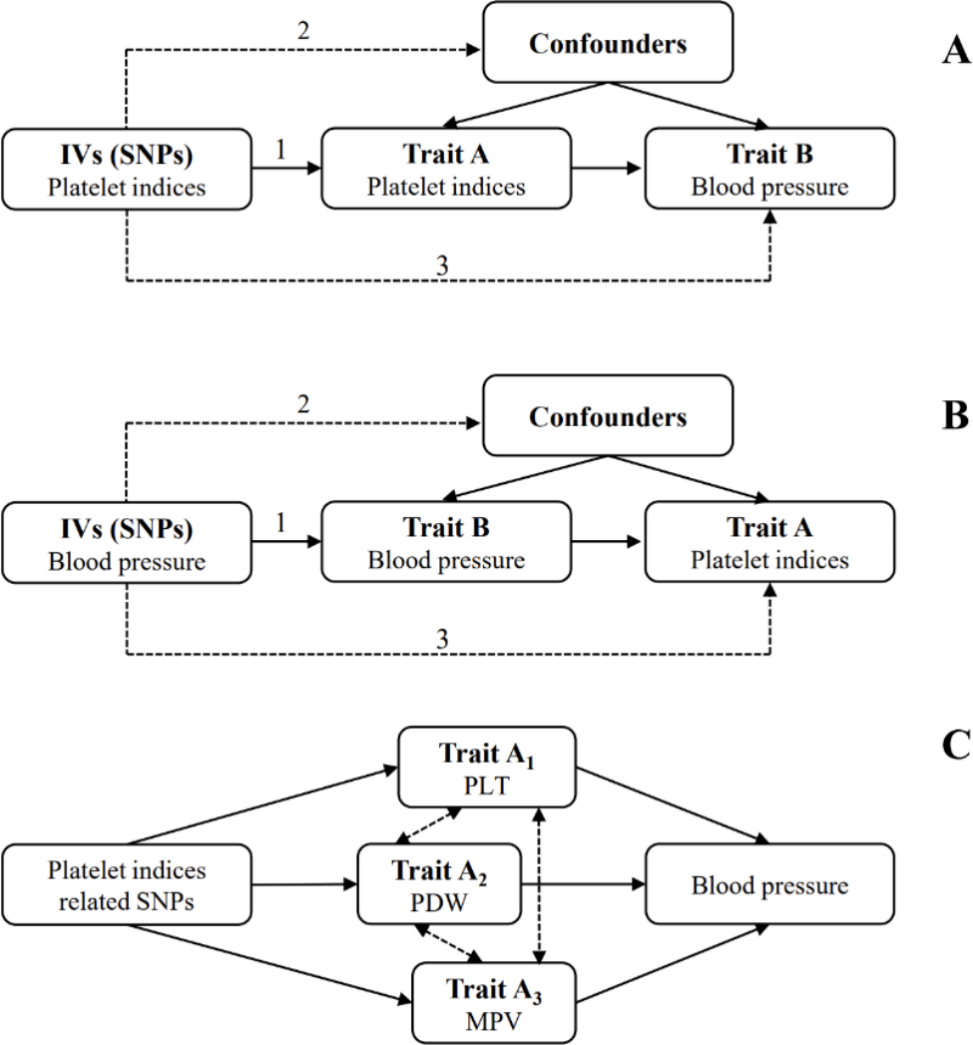
Directed acyclic graphs depicting the effects between platelet indices and BP. The analysis undertaken in this study used the MR method with 3 key assumptions satisfied. **A**: Platelet index SNPs were used as genetic instruments to investigate the causal effect of platelet indices on BP. **B**: BP SNPs were used as genetic instruments to investigate the causal effect of BP on platelet indices. Dashed lines indicate that SNPs are independent of any confounding variables between the results. **C**: Multivariable MR was applied to estimate the effect of platelet indices on BP. Dashed lines indicate that the traits are correlated (model 2).

## Materials and methods

### Study design and data source

The study design and data sources used are displayed in Supplementary Table 1. The genetic instruments for four platelet indices (PLT, PCT, MPV, and PDW) were selected from a GWAS conducted in the UK Biobank, which included 479,257 participants [28]. The mean (standard deviation [SD]) levels were 252.025 (60.061) × 10^9^ cells/Litre for PLT, 0.232 (0.049) % for PCT, 9.323 (1.085) femtolitres for MPV, and 16.496 (0.534) % for PDW. In addition, data on systolic blood pressure (SBP) and diastolic blood pressure (DBP) were also available from a published meta-analysis of the International Consortium of Blood Pressure (ICBP) and UK Biobank studies (Supplementary Table 2), which included 757,601 participants of European ancestry [29]. These selections were subjected to an MR analysis and met three key assumptions (Fig. 1): (1) the instrumental variables are directly related to the exposure of interest; (2) the instrumental variables are independent of any confounders; and (3) the genetic variants only affected outcomes via exposure [30]. The univariable MR analysis aimed to investigate the association of individual platelet indices with BP, and the multivariable MR analysis aimed to compare the independent effects of correlated indices on BP. Finally, to explore the possibility of reverse causation, we conducted an inverse MR analysis in which BP was treated as the exposure, and platelet indices were treated as the outcome. No ethical approval was required for this study, as the Biobank study in the UK was approved by the North West Multicentre Research Ethics Committee, and the initial studies included in the International Consortium of Blood Pressure (ICBP) were approved by an appropriate institutional review board.

### Selection of instrumental variables

Instrument variables (IVs) for each exposure considered in univariable MR analyses were selected as independent single-nucleotide polymorphisms (SNPs) at genome-wide significance (P < 5 × 10^− 8^). To identify independent SNPs, i.e., pairwise linkage disequilibrium (LD), we used thresholds of r^2^ (< 0.001, clumping window = 10,000 kbp) and a minor allele frequency threshold of 0.01. The selection of instruments for MVMR analyses was similar, and all SNPs with genome-wide significance were pooled and clumped to pairwise LD r^2^ < 0.001 based on the lowest P value for their association with any trait. Clumping was performed using the TwoSampleMR package in R. In regard to the second MR assumption, we inquired about each IV and its proxied features, referring to the PhenoScannerV2 database (http://www.phenoscanner.medschl.cam.ac.uk/) to identify whether there were SNPs associated with confounding factors (P < 5 × 10^− 8^). Palindromic variants were excluded from the primary analysis, and proxies were not used. MR pleiotropy residual sum and outlier (MR-PRESSO) analyses were performed to detect and remove outlier instruments. To avoid weak instrument bias, the F-statistic was measured to evaluate the total strength of the selected SNPs (F = R^2^ (n − 2)/(1 − R^2^), where R^2^ is the proportion of variance of the trait explained by the genetic instruments, and F > 10 is necessary for the complete set of SNPs). The IV ensembles and their R^2^, as well as the F-statistics used in the univariable MR analyses, are provided in Supplementary Table 3.

### Statistical analysis

Once we determined the IV sets based on the above selection criteria, we performed forwards MR analyses to estimate the overall effects. The IVW method was used as a significance analysis. This method provides an estimate with the highest power and relies on the assumption that all SNPs are valid instrumental variables. Secondary analyses were performed using the weighted median approach and MR‒Egger regression to examine the robustness of the findings and adjust for pleiotropy. The MR‒Egger approach allows for the detection and correction of directional pleiotropy, albeit with compromised power. Afterwards, we applied the same MR methods as above in reverse direction MR analysis. Sensitivity analyses were performed to test the sensitivity of the disproportionate effects of variants and pleiotropy using the leave-one-out method. To ascertain the presence of pleiotropy, we additionally applied outlier (MRPRESSO) analysis. Given genetic and phenotypic correlations across platelet indices, we used the multivariable IVW method to disentangle and compare the effects of correlated platelet indices on BP. Finally, we performed collinearity assays with the lasso method and readjusted the MVMR results. Effect estimates were reported in beta (for a one-SD increase in exposure) with a 95% confidence interval (CI). In the univariable MR analysis, the results of the causal effect were considered to have statistical significance when P < 0.006 (Bonferroni correction) to represent strong evidence of causal associations, given the issue of multiple testing. The associations with P values below 0.05 but above 0.006 were considered suggestive evidence of associations. The sensitivity testing and the P value adjustments were not tailed for multivariable MR analysis due to the mutual adjustment nature of multivariable MR analysis. The results of sensitivity analyses regarding the causal effects of exposures and outcomes were considered statistically significant when P < 0.05. Given potential sample overlap, statistical bias was estimated using a web tool (https://sb452.shinyapps.io/overlap/) [31], and the results are shown in Supplementary Table 4. All statistical analyses were conducted using R statistical software with the TwoSampleMR and MRPRESSO Packages (version 4.2.0, R Foundation for Statistical Computing, Vienna, Austria, 2022; https://www.R-project.org).

**Fig. 2:**
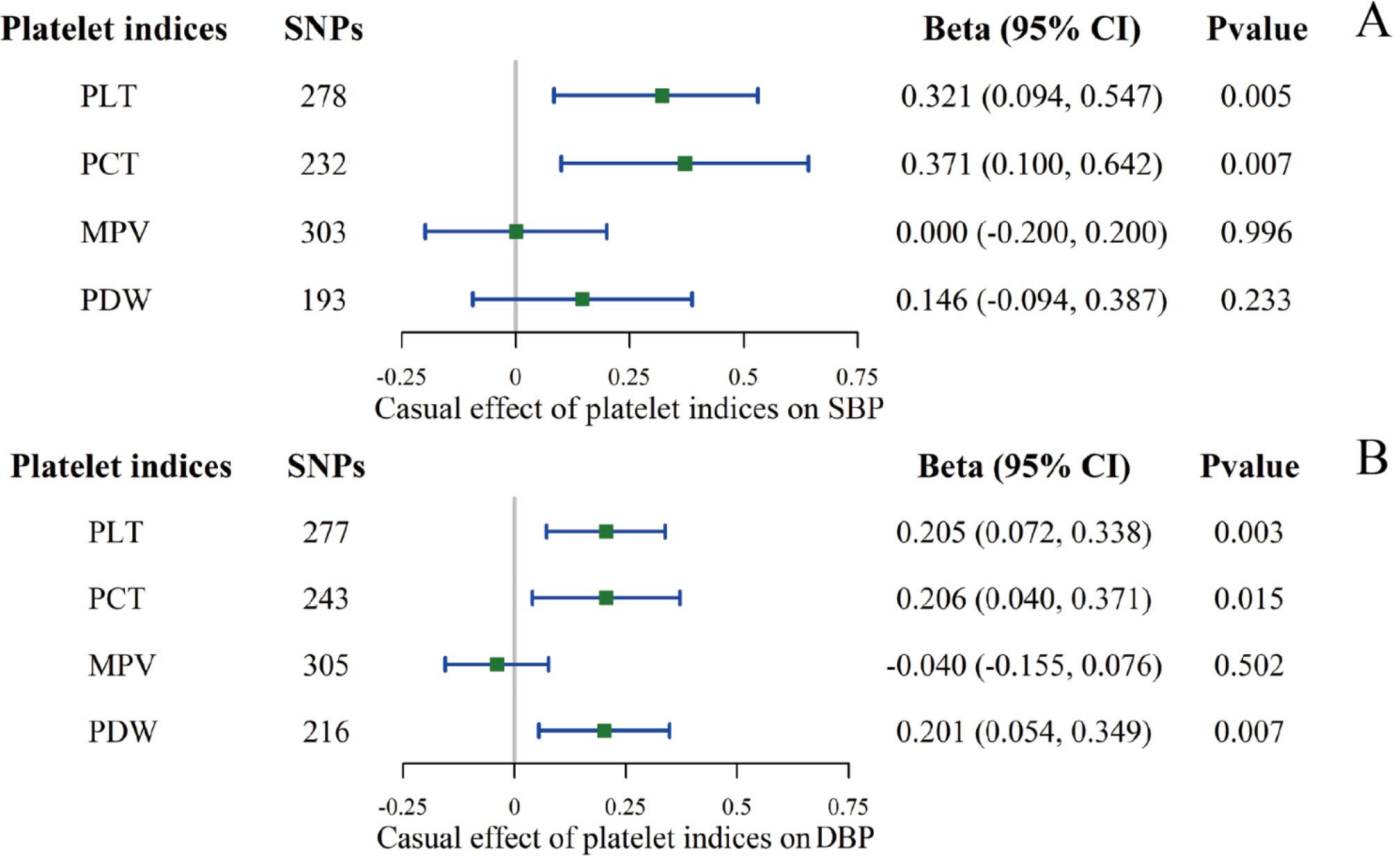
The causal effect of platelet indices on BP using the IVW method in univariable MR. **A**: to SBP; **B**: to DBP. The causal effect was expressed as beta (for a one-SD increase in exposure) and 95% CIs of the estimates

**Fig. 3:**
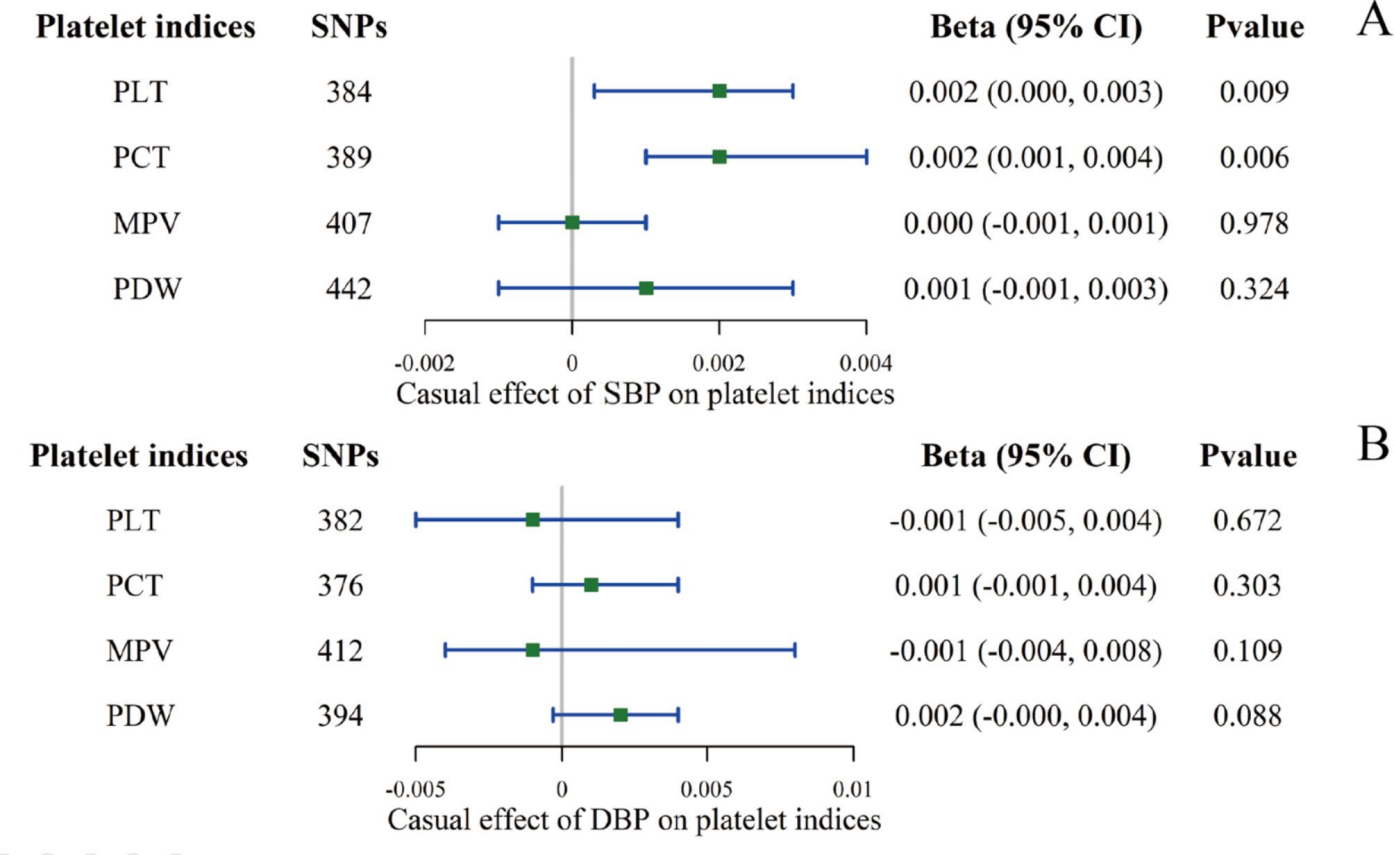
The causal effect of BP on platelet indices using the IVW method in univariable MR. **A**: effect of SBP on platelet indices; **B**: effect of DBP on platelet indices. The causal effect was expressed as beta (for a one-SD increase in exposure) and 95% CIs of the estimates

## Results

**Fig. 4:**
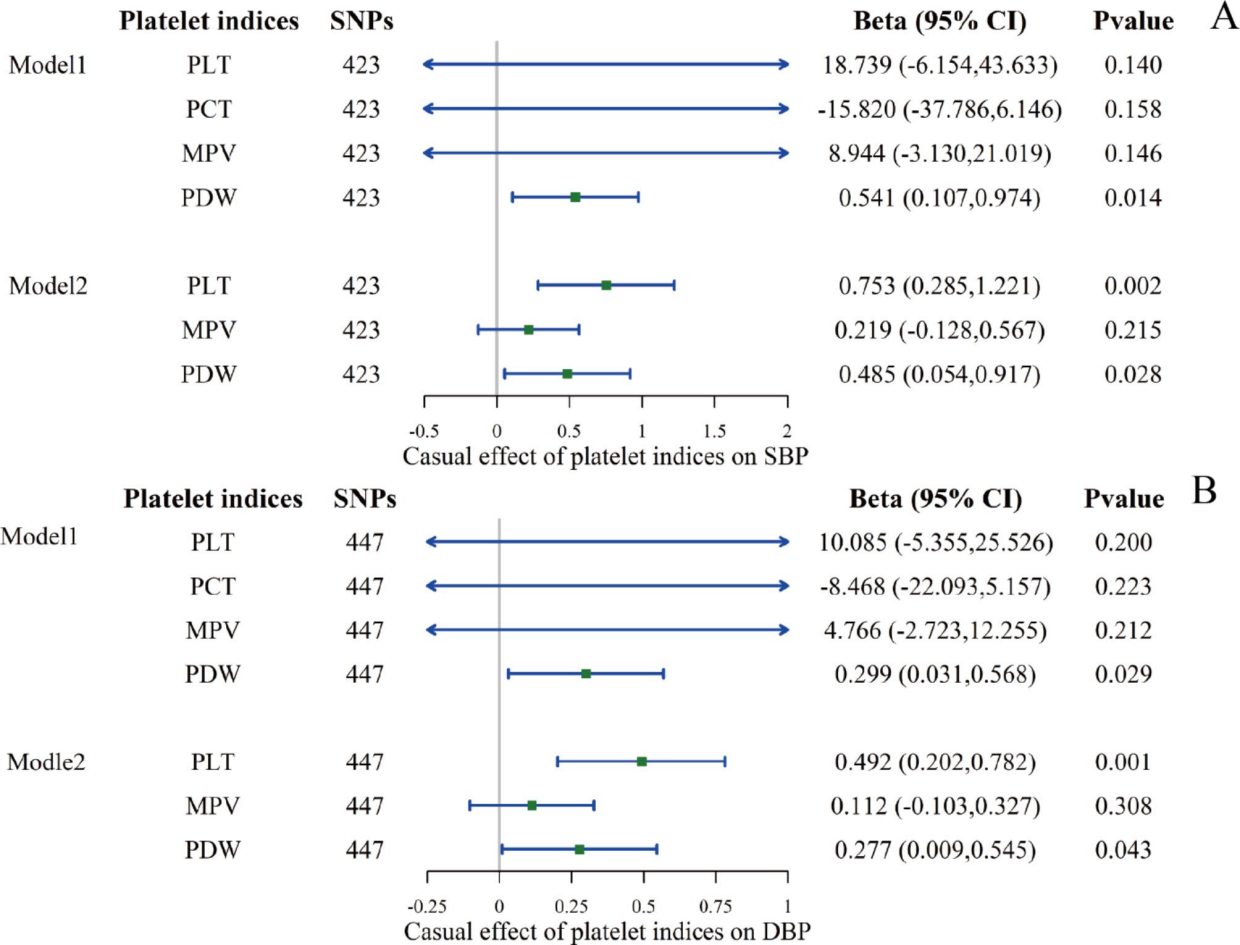
The causal effect of platelet indices on BP using the IVW method in multivariable MR. **A**: on SBP; **B**: on DBP. Four platelet indices were mutually adjusted in Model 1, and in Model 2, three indices were mutually adjusted except PCT after colinear correction. The causal effect was expressed as beta (for a one-SD increase in exposure) and 95% CIs of the estimates

All genetic association estimates in the univariable MR analyses are provided in Supplementary Tables 5 and visualized in Figs. 2 and 3. For the primary IVW MR analyses after the removal of outliers, we found evidence for a positive effect of the genetically predicted value of PLT, PCT, and PDW on the level of DBP (PLT: beta 0.205, 95% CI 0.072–0.338, P = 0.003; PCT: beta 0.206, 95% CI 0.040–0.371, P = 0.015; PDW: beta 0.201, 95% CI 0.054–0.349, P = 0.007). PLT and PCT maintained their roles in SBP (PLT: beta 0.321, 95% CI 0.094–0.547, P = 0.005; PCT: beta 0.371, 95% CI 0.100–0.642, P = 0.007), but the effect of PDW was eliminated. Reverse MR was conducted to evaluate whether elevated BP was associated with the activation of platelets, as reflected by platelet indices. No platelet traits were influenced by BP, except that SBP had a negligible impact on PLT and PCT in the IVW (PLT: beta 0.002, 95% CI: 0.000–0.003, P = 0.009; PCT: beta 0.002, CI: 0.001–0.004, P = 0.006). Sensitivity analyses were performed as described in Supplementary Table 6. The MR‒Egger test of the intercepts did not provide evidence of directional pleiotropy for the exposure, and the global MR-PRESSO tests were all nonsignificant after removing the outliers. Heterogeneity, however, was significant according to the results of the Cochran’s Q test. The results of the multivariable MR analysis are displayed in Fig. 4 and Supplementary Tables 7 and 8. PDW retained a robust causal association with DBP elevation after mutual adjustment for four platelet indices, and its effect on SBP re-emerged. Based on the potential collinearity problem, we performed the lasso test (collinearity correction). After excluding PCT, PLT’s independent effect on BP was again essential (on SBP: beta 0.753, 95% CI: 0.285–1.221, P = 0.002; on DBP: beta 0.002, CI: 0.202–0.782, P = 0.001).

## Discussion

We used IVs of the four platelet indices and BP in the current study to explore their association. In the univariable MR, we found that the levels of PLT and PCT predicted by host genetics were causally associated with an increase in BP (SBP, DBP). PDW was associated with DBP, and the magnitude of its association with SBP was similar but nonsignificant. Univariable MR rules out external interference, but it still fails to disentangle and compare the effects of correlated platelet indices on BP. In MVMR, the relationship between PWD and SBP was again significant, which could be the effect of PDW was concealed by other indices in the univariable MR model. The effect of PLT and PDW on BP remained robust after correcting for collinearity in MVMR (Model 2), whereas PLT and PCT associations with BP became non-significant. These findings suggest that PLT and PDW are the critical traits underlying the positive associations of platelet activation with elevated BP. In the reverse MR, we observed a negligible effect of SBP on PLT and PCT. It means that even a considerable increase in SBP may result in only subtle changes in PCT and PLT. It is worth noting that the findings should not be extrapolated to the extremes, as no amount of BP in humans can increase both PCT and PLT to a significant degree. However, small (beta value) does not imply zero, the p-values were statistically significant suggesting a causal relationship.

Several studies have reported that platelet activation is related to cardiovascular morbidity and mortality [23, 32]. Platelet indices are blood-based parameters related to platelet morphology and proliferation kinetics, and they can be used to reflect the activation of platelets. Increased platelet indices were observed in multiple diseases, such as ischaemic heart disease and ischaemic strokes [19, 24] [33, 34]. So the pathway from platelet activation to changes in platelet indices have been established. In this context, we can theorize that BP level changes when platelets are activated to cause changes in platelet indices. Our findings of the univariable MR study align with earlier studies on PLT concerning the elevation of BP [14, 22]. To the best of our knowledge, no MR study has evaluated the effect of the other platelet indices on BP, whereas the association of hypertension with those indices has been reported in observational studies [14–16]. Some studies have also shown that MPV is associated with the incidence of hypertension. Nevertheless, it is worth noting that the conclusions from observational studies are prone to be biased by confounding effects and reverse causation. Our study found no association between MPV and BP, except for PCT.

Studies on the comparative effects of platelet indices on BP are limited. Given the coassociation among platelet indices, it is necessary to explore which trait or traits predominate the influence of platelets on BP. We then performed MVMR to disentangle the comparative effects of platelet indices. The present MR study expanded the preexisting evidence to show that PLT alone with PDW exhibited independent effects. PLT, as is commonly known, measures platelet counts per unit volume of blood, while PCT is the volume occupied by platelets in blood expressed as a percentage and MPV is a marker of the average size of platelets. Therefore, PCT is analogous to the total platelet volume in some way and is correlated to the product of MPV and PLT. The results of MVMR excluding PCT after the colinear test provide evidence that heterogeneity of platelet size, i.e., PDW, is a marker of more significant importance among PCT and MPV. Therefore, high PLT and PDW might be an indication to recognize the role of platelet activation in interpreting the process of hypertension.

In in vitro studies, the increased shear force that platelets are exposed to due to high BP could lead to platelet activation, and the giant platelets are more aggregated and reactive than the smaller platelets [35, 36]. In the reverse direction, the present study found that SBP influences PLT and PCT. However, a previous MR study based on a smaller sample size revealed that hypertension has no effect on PLT [22]. The discrepancy might be caused by inadequate power due to limited phenotypic variance explained by the genetic variants used and/or small sample size for outcomes. Nevertheless, whether BP has effects on platelet activation needs more study.

The benefit obtained from a reduction in BP is undisputed [37, 38]. So the findings of our study are relevant in both clinical and public health terms. We revealed that platelet activation is one of the causes of elevated BP and that it is mirrored by platelet indices, which are potentially valuable markers for evaluating hypertension progression and the early diagnosis of thromboembolic disease. Among the four platelet indices, PLT and PDW played independent and dominant roles, which should be emphasized in clinical practice. As previous studies have shown that platelet activation plays a significant role in embolic disease; the use of antiplatelet management therapies in individuals with high BP should be considered [12, 39]. However, there is a tendency to ignore this primary prevention, and a great deal of attention has been paied to the re-prevention after cardiovascular events. In this study, we give the evidence that antiplatelet therapy in people with high BP is reasonable, so the primary prevention of CVDs in hypertension group should be emphasized. The latest review showed no evidence that antiplatelet therapy modifies mortality in patients with elevated BP for primary prevention [13]. The reason for this result is that bleeding events outweighed the potential benefits. However, this does not mean that antiplatelet therapy is worthless for people with hypertension. In contrast, small doses of antiplatelet therapy may benefit a specific population if BP is controlled smoothly and the risk of bleeding and embolism is accurately assessed. Overall, further randomized controlled trials (RCTs) assessing antithrombotic therapy with complete documentation of all benefits and harms are required in patients with elevated BP.

A consistent limitation of all the discussed observational studies is that platelet indices are influenced by various conditions, which can confound the analysis. Therefore, the strengths of our study are that this potential source of bias was overcome through the use of genetic variants as instruments for platelet indices and the possibility of reverse causation was explored [28, 29, 40]. Furthermore, the multivariable MR method was the major strength, which compared the roles of different correlated platelet traits in BP elevation. We also implemented a rigorous methodological approach, incorporating a range of sensitivity analyses to explore potential bias due to genetic pleiotropy.

This study also has limitations. First, the GWAS of traits associated with platelets was performed in the UK Biobank population, and BP data were available from meta-analyses published by the International Consortium of Blood Pressure (ICBP) and UK Biobank studies. Therefore, there is the potential for overlap in samples and bias related to this fact [31]. Second, we added a more significant number of SNPs as IVs with no horizontal pleiotropy to increase the R^2^. As a result, the improvement of SNPs in the instrument came increasingly due to heterogeneity. Third, we only included four platelet indices in the study; there may be other hematological markers related to platelets, which means that the dominant role of PLT and PDW could need adjustment when including other traits, such as platelet-large cell ratio (P-LCR).

MR assumes a linear relationship between the exposure and outcome [41], which, in our case, included genetically determined platelet indices and BP level. For this reason, the results of our MR analysis should not be extrapolated to extremes of platelet indices and BP. Of particular note is the possibility that platelets may be a causal risk factor for the progression rather than the onset of hypertension; there could be distinct causes for the initiation and progression of a disease, which means that the causal exposures for disease onset may not necessarily be causal for disease progression (and vice versa) [42].

## Conclusions

The current MR study provides evidence in support of PLT and PDW as independent and predominant traits accounting for the basis of PLT, PCT, MPV, and PDW in relation to BP levels. Conversely, SBP has a weak influence on PLT and PCT. In summary, we extended the evidence that platelets may not only be bystanders in the pathogenesis and progression of hypertension. Understanding the role of platelets and their related traits is valuable for clinical as well as public health. What is not established is the direct effect of platelet activation on BP, so future basic experimental studies should explore specific pathophysiologic mechanisms. Stratified RCT research is also needed to give antiplatelet therapy more evidence as the primary prevention of thrombotic disease in people with high BP.

## Electronic supplementary material

Below is the link to the electronic supplementary material.

Supplementary Material 1

## Data Availability

All summary datasets analysed during the current study are publicly available.

## Abbreviations

BP: Blood pressure
CI: Confidence interval
DBP: Diastolic blood pressure
GWAS: Genome-wide association studies
IVW: Inverse variance weighting
LD: Linkage disequilibrium
MPV: Mean platelet volume
MR: Mendelian randomization
MVMR: Multivariable Mendelian randomization
PCT: Plateletcrit
PDW: Platelet distribution width
PLT: Platelet count
PRESSO: Pleiotropy residual sum and outlier
RCT: Randomized controlled trial
SBP: Systolic blood pressure
SD: Standard deviation
SNP: Single nucleotide polymorphism
