# Supplementary material for: Platelet indices and blood pressure: a multivariable mendelian randomization study
Source: Thromb J. 2023 Mar 20;21:31. doi: 10.1186/s12959-023-00475-6 (PMC10026509; doi:10.1186/s12959-023-00475-6)
Supplement: Supplementary file 1 — Supplementary Material 1 [file 12959_2023_475_MOESM1_ESM.docx]

**Supporting information**

**Platelet indices and blood pressure: A Multivariable Mendelian Randomization Study**

**Supplementary table 1**. Detailed information on used studies

**Supplementary table 2**. Basic characteristics of the studies that contributed summary statistics of blood pressure (ICBP-UK Biobank)

**Supplementary table 3**. Genetic instruments selected for exposure

**Supplementary table 4**. Bias and type 1 error rate for Mendelian randomization with sample overlap

**Supplementary table 5.** Causal estimation between platelet indices and blood pressure in bidirectional univariable MR analysis

**Supplementary table 6.** Associations of platelet indices with blood pressure in sensitivity analyses

**Supplementary table 7.** Associations of platelet indices with blood pressure in MVMR

**Supplementary table 8.** Associations of platelet indices with blood pressure in MVMR after co-linearity correction

**Supplementary table 1**. Detailed information on used studies

| **Trait** | **Consortium or cohort study** | **Number of participants included in genotyping** | **PubMed ID or web source** | **GWAS ID** |
| --- | --- | --- | --- | --- |
| PLT | UK Biobank | 350 474 | 34594039 | ukb-d-30080_irnt |
| PCT | UK Biobank | 350 471 | <http://www.nealelab.is/> | ukb-d-30090_irnt |
| MPV | UK Biobank | 350 470 |  | ukb-d-30100_irnt |
| PDW | UK Biobank | 350 470 |  | ukb-d-30110_irnt |
| SBP  DBP | UK Biobank+ ICBP  UK Biobank+ ICBP | 757 610  757 610 | 30224653 | ieu-b-38  ieu-b-39 |
|  |  |  | 30224653 |  |

DBP, diastolic blood pressure; ICBP, International Consortium of Blood Pressure; MPV, mean platelet volume; PLT platelet count; PCT, plateletcrit; PDW, platelet distribution width; SBP, systolic blood pressure

**Supplementary table 2**. Basic characteristics of the studies that contributed summary statistics of blood pressure (ICBP-UK Biobank)

| **Characteristic *** | **ICBP** | **UKB** |
| --- | --- | --- |
|  |  |  |
| **Sample size**  **Age(yr),mean**  **Male percentage**  **SBP (mmHg),mean** | 299,024  54.9  44.9  134.3 | 458,577  56.8  45.8  141.1 |
| **DBP (mmHg)** | 80.6 | 84.3 |

DBP, diastolic blood pressure; ICBP, International Consortium of Blood Pressure; SBP, systolic blood pressure

**^*^** The basic characteristics of the studies participated in the ICBP were calculated as the weighted average of the characteristics of each study, with the study-specific sample size as the weight. These study-specific characteristics and the UK Biobank characteristics were reported in Evangelou E, Warren HR, Mosen-Ansorena D, et al. Genetic analysis of over 1 million people identifies 535 new loci associated with blood pressure traits. Nat Genet. 2018;50:1412–1425.

**Supplementary table 3.** Genetic instruments selected for exposure

| **Exposure** | **Outcome** | **SNPs** | **R^2^** | **F statistic** |
| --- | --- | --- | --- | --- |
| PLT | SBP | 278 | 0.046 | 59 |
| PCT | SBP | 232 | 0.022 | 35 |
| MPV | SBP | 303 | 0.119 | 149 |
| PDW | SBP | 193 | 0.030 | 45 |
| PLT | DBP | 277 | 0.036 | 47 |
| PCT | DBP | 243 | 0.024 | 35 |
| MPV | DBP | 305 | 0.109 | 143 |
| PDW | DBP | 216 | 0.031 | 50 |
| SBP | PLT | 384 | 0.022 | 42 |
| SBP | PCT | 389 | 0.022 | 41 |
| SBP | MPV | 407 | 0.021 | 38 |
| SBP | PDW | 442 | 0.022 | 39 |
| DBP | PLT | 382 | 0.023 | 45 |
| DBP | PCT | 376 | 0.022 | 43 |
| DBP | MPV | 412 | 0.020 | 40 |
| DBP | PDW | 394 | 0.027 | 48 |

DBP, diastolic blood pressure; MPV, mean platelet volume; PLT platelet count; PCT, plateletcrit; PDW, platelet distribution width; SBP, systolic blood pressure; SNPs, single-nucleotide polymorphisms.

**Supplementary table 4**. Bias and Type 1 error rate for Mendelian randomization with sample overlap

| **Exposure** | **Outcome** | **Bias (type 1 error rate) of overlap proportion** | | | | | |
| --- | --- | --- | --- | --- | --- | --- | --- |
|  |  | **0.0** | **0.1** | **0.2** | **0.3** | **0.4** | **0.5** |
| PLT | SBP | 0.000(0.05) | 0.000(0.05) | 0.001(0.05) | 0.001(0.05) | 0.001(0.06) | 0.002(0.06) |
| PCT | SBP | 0.000(0.05) | 0.001(0.05) | 0.001(0.05) | 0.002(0.06) | 0.002(0.06) | 0.003(0.07) |
| MPV | SBP | 0.000(0.05) | 0.000(0.05) | 0.000(0.05) | 0.000(0.05) | 0.001(0.05) | 0.001(0.05) |
| PDW | SBP | 0.000(0.05) | 0.000(0.05) | 0.001(0.05) | 0.001(0.05) | 0.002(0.06) | 0.002(0.06) |
| PLT | DBP | 0.000(0.05) | 0.000(0.05) | 0.001(0.05) | 0.001(0.06) | 0.002(0.06) | 0.002(0.06) |
| PCT | DBP | 0.000(0.05) | 0.001(0.05) | 0.001(0.05) | 0.002(0.06) | 0.002(0.06) | 0.003(0.07) |
| MPV | DBP | 0.000(0.05) | 0.000(0.05) | 0.000(0.05) | 0.000(0.05) | 0.001(0.05) | 0.001(0.05) |
| PDW | DBP | 0.000(0.05) | 0.000(0.05) | 0.001(0.05) | 0.001(0.05) | 0.002(0.06) | 0.002(0.06) |
| SBP | PLT | 0.000(0.05) | 0.000(0.05) | 0.000(0.05) | 0.000(0.05) | 0.000(0.05) | 0.000(0.05) |
| SBP | PCT | 0.000(0.05) | 0.000(0.05) | 0.000(0.05) | 0.000(0.05) | 0.000(0.05) | 0.000(0.05) |
| SBP | MPV | 0.000(0.05) | 0.000(0.05) | 0.000(0.05) | 0.000(0.05) | 0.000(0.05) | 0.000(0.05) |
| SBP | PDW | 0.000(0.05) | 0.000(0.05) | 0.000(0.05) | 0.000(0.05) | 0.000(0.05) | 0.000(0.05) |
| DBP | PLT | 0.000(0.05) | 0.000(0.05) | 0.000(0.05) | 0.000(0.05) | 0.000(0.05) | 0.000(0.05) |
| DBP | PCT | 0.000(0.05) | 0.000(0.05) | 0.000(0.05) | 0.000(0.05) | 0.000(0.05) | 0.000(0.05) |
| DBP | MPV | 0.000(0.05) | 0.000(0.05) | 0.000(0.05) | 0.000(0.05) | 0.000(0.05) | 0.000(0.05) |
| DBP | PDW | 0.000(0.05) | 0.000(0.05) | 0.000(0.05) | 0.000(0.05) | 0.000(0.05) | 0.000(0.05) |

Bias estimation was calculated using a webtool (<https://sb452.shinyapps.io/overlap/>).

DBP, diastolic blood pressure; MPV, mean platelet volume; MVMR multivariable Mendelian randomization; PLT platelet count; PCT, plateletcrit; PDW, platelet distribution width; SBP, systolic blood pressure.

**Supplementary table 5.** Causal estimation between platelet indices and blood pressure in bidirectional univariable MR analysis

| **Exposure** | **Outcome** | **Weighted median method** | | | **MR-Egger regression** | |  | **Inverse variance weighted** | | |
| --- | --- | --- | --- | --- | --- | --- | --- | --- | --- | --- |
|  |  | **Beta** | **95% CI** | ***p*** | **Beta** | **95% CI** | ***p*** | **Beta** | **95%CI** | ***p*** |
| PLT | SBP | 0.398 | 0.149,0.647 | 0.002 | 0.094 | -0.306,0.496 | 0.643 | 0.321 | 0.094,0.547 | 0.005 |
| PCT | SBP | 0.176 | -0.151,0.502 | 0.032 | 0.614 | 0.056,1.172 | 0.031 | 0.371 | 0.100,0.642 | 0.007 |
| MPV | SBP | -0.142 | -0.324,0.038 | 0.123 | -0.024 | -0.317,0.269 | 0.872 | 0.000 | -0.200,0.200 | 0.996 |
| PDW | SBP | 0.018 | -0.257,0.293 | 0.896 | 0.077 | -0.351, 0.505 | 0.723 | 0.146 | -0.094,0.387 | 0.233 |
| PLT | DBP | 0.248 | 0.077,0.419 | 0.004 | 0.291 | 0.047,0.536 | 0.020 | 0.205 | 0.072,0.338 | 0.003 |
| PCT | DBP | 0.154 | -0.039,0.347 | 0.117 | 0.292 | -0.052,0.636 | 0.097 | 0.206 | 0.040, 0.371 | 0.015 |
| MPV | DBP | -0.106 | -0.213,-0.001 | 0.049 | -0.076 | -0.247,0.094 | 0.379 | -0.040 | -0.155,0.076 | 0.502 |
| PDW | DBP | 0.215 | 0.058,0.373 | 0.007 | 0.283 | 0.022,0.545 | 0.565 | 0.201 | 0.054, 0.349 | 0.007 |
| SBP | PLT | 0.000 | -0.001,0.002 | 0.536 | 0.001 | -0.002,0.005 | 0.476 | 0.002 | 0.000,0.003 | 0.009 |
| SBP | PCT | 0.001 | -0.001,0.004 | 0.330 | 0.002 | -0.002,0.005 | 0.358 | 0.002 | 0.001,0.004 | 0.006 |
| SBP | MPV | 0.000 | -0.002,0.002 | 0.911 | 0.001 | -0.005,0.002 | 0.406 | <0.001 | -0.001,0.001 | 0.978 |
| SBP | PDW | -0.001 | -0.003,0.001 | 0.241 | 0.000 | -0.006,0.005 | 0.954 | 0.001 | -0.001,0.003 | 0.324 |
| DBP | PLT | 0.001 | -0.002,0.004 | 0.687 | 0.003 | -0.009,0.014 | 0.651 | -0.001 | -0.005,0.004 | 0.672 |
| DBP | PCT | 0.000 | -0.003,0.004 | 0.968 | 0.004 | 0.002,0.010 | 0.221 | 0.001 | -0.001,0.004 | 0.303 |
| DBP | MPV | 0.001 | -0.002,0.004 | 0.422 | 0.007 | -0.004,0.018 | 0.255 | -0.001 | -0.004,0.008 | 0.109 |
| DBP | PDW | -0.003 | -0.006,0.000 | 0.079 | -0.003 | -0.009,0.004 | 0.430 | 0.002 | -0.000,0.004 | 0.088 |

CI indicates confidence interval; DBP, diastolic blood pressure; MPV, mean platelet volume; MVMR multivariable Mendelian randomization; PLT platelet count; PCT, plateletcrit; PDW, platelet distribution width; SBP, systolic blood pressure; SNPs, single-nucleotide polymorphisms.

**Supplementary table 6.** Associations of platelet indices with blood pressure in sensitivity analyses

| **Exposure** | **Outcome** | **I2(%)** | **MR-Egger intercept** | **P of MR-Egger intercept** |  | **P of Cochran's Q heterogeneity test** | **P of MR‐PRESSO global test** | |  |
| --- | --- | --- | --- | --- | --- | --- | --- | --- | --- |
| PLT | SBP | 64 | 0.009 | 0.181 | | <0.001 | 0.982 |  | |
| PCT | SBP | 60 | -0.007 | 0.329 |  | <0.001 | 0.392 |  | |
| MPV | SBP | 79 | 0.001 | 0.822 |  | <0.001 | 0.626 |  | |
| PDW | SBP | 70 | 0.003 | 0.702 |  | <0.001 | 0.750 |  | |
| PLT | DBP | 65 | -0.003 | 0.412 |  | <0.001 | 0.983 |  | |
| PCT | DBP | 66 | -0.002 | 0.575 |  | <0.001 | 0.392 |  | |
| MPV | DBP | 81 | 0.002 | 0.052 |  | <0.001 | 0.612 |  | |
| PDW | DBP | 69 | -0.003 | 0.456 |  | <0.001 | 0.862 |  | |
| SBP | PLT | 67 | 0.000 | 0.071 |  | <0.001 | 0.213 |  | |
| SBP | PCT | 68 | 0.000 | 0.822 |  | <0.001 | 0.308 |  | |
| SBP | MPV | 61 | 0.001 | 0.362 |  | <0.001 | 0.937 |  | |
| SBP | PDW | 86 | 0.000 | 0.623 |  | <0.001 | 0.145 |  | |
| DBP | PLT | 89 | -0.001 | 0.494 |  | <0.001 | 0.213 |  | |
| DBP | PCT | 65 | -0.001 | 0.380 |  | <0.001 | 0.302 |  | |
| DBP | MPV | 89 | -0.001 | 0.595 |  | <0.001 | 0.498 |  | |
| DBP | PDW | 64 | 0.001 | 0.114 |  | <0.001 | 0.763 |  | |

DBP, diastolic blood pressure; MPV, mean platelet volume; PLT platelet count; PCT, plateletcrit; PDW, platelet distribution width; SBP, systolic blood pressure.

**Supplementary table 7.** Associations of platelet indices with blood pressure in MVMR

| **Exposure** | **Outcome** | **SNPs** | **Beta** | **95% CI** | ***p*** |
| --- | --- | --- | --- | --- | --- |
| PLT  PCT  MPV  PDW  PLT  PCT  MPV  PDW | SBP  SBP  SBP  SBP  DBP  DBP  DBP  DBP | 423  423  423  423  447  447  447  447 | 18.739  -15.820  8.944  0.541  10.085  -8.468  4.766  0.299 | -6.154,43.633  -37.786,6.146  -3.130,21.019  0.107,0.974  -5.355,25.526  -22.093,5.157  -2.723,12.255  0.031,0.568 | 0.140  0.158  0.146  0.014  0.200  0.223  0.212  0.029 |

DBP, diastolic blood pressure; MPV, mean platelet volume; MVMR multivariable Mendelian randomization; PLT platelet count; PCT, plateletcrit; PDW, platelet distribution width; SBP, systolic blood pressure.

**Supplementary table 8.** Associations of platelet indices with blood pressure in MVMR after co-linearity correction

| **Exposure** | **Outcome** | **SNPs** | **Beta** | **95% CI** | ***p*** |
| --- | --- | --- | --- | --- | --- |
| PLT  MPV  PDW  PLT  MPV  PDW | SBP  SBP  SBP  DBP  DBP  DBP | 423  423  423  447  447  447 | 0.753  0.219  0.485  0.492  0.112  0.277 | 0.285,1.221  -0.128,0.567  0.054,0.917  0.202,0.782  -0.103,0.327  0.009,0.545 | 0.002  0.215  0.028  0.001  0.308  0.043 |

DBP, diastolic blood pressure; MPV, mean platelet volume; MVMR multivariable Mendelian randomization; PLT platelet count; PDW, platelet distribution width; SBP, systolic blood pressure.
